# Language introduction as a space for the inclusion and exclusion of young asylum seekers in Sweden

**DOI:** 10.1080/17482631.2020.1761196

**Published:** 2020-12-09

**Authors:** Andreas Fejes, Magnus Dahlstedt

**Affiliations:** a Department of Behavioural Sciences and Learning, Linköping University, Linköping, Sweden; b Department of Culture and Society, Linköping University, Norrköping, Sweden

**Keywords:** Asylum seekers, language introduction, folk high schools, Upper secondary school, inclusion and exclusion

## Abstract

**Purpose**: In this article, we focus on the language introduction (LI) programme for newly arrived migrants aged 16–19 in Sweden. We ask how it is organized for inclusion and what kinds of exclusion arise from such an organization. More specifically, we ask the question: in what ways do different settings for LI exclude at the same time as they include?

**Method**: Drawing on Lister’s approach to inclusion and exclusion, we analyse interviews with students, teachers and principals at five schools that deliver the LI programme: two municipality-run upper secondary schools, two folk high schools, and one independent upper secondary school.

**Result**: We illustrate that the question of inclusion and exclusion is not a matter of either/or. Rather, these processes coexist and include several dimensions, including rights and responsibilities, participation, and belonging.

**Conclusion**: We argue that, in order to create an understanding of school as a place for inclusion (and exclusion), we need a broad approach that makes it possible to identify these different dimensions, and how they are related.

## Introduction

Over the past few years, Sweden has received one of the highest rates of asylum seekers per capita in Europe, not least involving Syrian refugees. Among those seeking refuge in Sweden are a large number of children and young adults, many of whom are unaccompanied. As is the case all across Europe, the migration situation has sparked intense debates concerning issues of migration and integration. Over a short period of time, the political climate has changed quite dramatically (Dahlstedt & Neergaard, 2019)—from a situation in which Sweden was internationally renowned for having developed one of the most generous models for the reception and inclusion of migrants, to one where calls are being made for more rigorous border controls, and for migrants to adapt to “Swedish values” (Schierup et al., 2017). Such calls have also been made in relation to young asylum seekers, particularly unaccompanied minors, and the range of “problems” they allegedly cause, and will cause in the future (Herz & Lalander, 2019; Nordling, 2017). They are construed as different, causing conflicts, and having problems in adapting to the new host society. Furthermore, the suspicion is cast on their motives for seeking asylum, especially in terms of being honest about their age.

At the same time, especially during 2015 and 2016, a range of initiatives and support structures were put in place to support new arrivals on their paths towards inclusion into society. Such support was provided by both state agencies and civil society organizations. Among these support structures, education is especially important. In this article, we will direct attention towards the main educational institution for young people in Sweden, upper secondary school, and specifically the language introduction programme (LI), aimed at asylum seekers aged 16–19. The focus here is on learning the Swedish language in order to become eligible for a regular national programme. The overall aim of this article is to examine how the LI programme is organized for inclusion and what kind of exclusions arise from such an organization.

## Previous research

As illustrated in previous studies on young migrant students in Sweden (cf. Jepson Wigg, 2008; Nilsson Folke, 2017; Sharif, 2017), as well as in other countries (Pinson & Arnot, 2010; Terhart & Devitz, 2018), the educational space becomes highly important to them, not only in terms of learning the language, but also as a social space. In this space, there are opportunities to meet other students, with different backgrounds, as well as to receive various kinds of support, from teachers and students alike (Rodríguez-Izquierdo & Darmody, 2019). For some students, teachers become important, sometimes being their only role models, because they have fled their country of origin on their own (Fejes et al., 2018a). However, despite the educational space playing such a role as a means of inclusion, it might at the same time be quite excluding (see, e.g. Nilsson Folke, 2017; Sharif, 2017; Torbjørnsen Hilt, 2016), not least due to the placement of newly arrived students in separate classes. Although such placement might provide students with a sense of community and belonging, as well as a feeling of the classroom being a safe space where all students face similar challenges, not least in terms of learning the Swedish language, the separation also creates a sense of shame and otherness (Nilsson Folke, 2017; Skowronski, 2013). When students are separated in this way, ethnocultural spaces emerge within schools, where newly arrived students are both physically and mentally shuffled “off to the side”, like a symbolic extension of an excluded position in Swedish society at large (Sharif, 2017).

Although a range of studies of LI or the equivalent has been carried out, in which issues of inclusion and exclusion have been central, they have usually focused on single schools, or schools run by municipalities. In this article, we direct our attention towards how LI is organized at five different schools, two ordinary municipality-run upper secondary schools, one independent upper secondary school, and two folk high schools. Thus, we will be able to further investigate whether there are any discernible differences in how practices of inclusion and exclusion emerge in different educational settings for LI.

## The language introduction programme

LI is one of the introductory programmes in Swedish upper secondary school, created in the wake of the upper secondary school reform of 2011. It targets newly arrived students, with the aim of teaching them the Swedish language to enable them to enter a national upper secondary programme (i.e. LI is comparable to compulsory school in terms of content). A student may enter LI no later than the first half of the year in which they turn 18. LI is part of the Swedish educational system, which is regulated by the State, with the municipalities having responsibility for providing and financing the education. However, for LI, there is no national knowledge requirement. Rather, municipalities have to locally formulate a plan for LI in which the aim, length, and content are stated. As well as such a plan, LI must follow the national syllabus for Swedish as a second language within the compulsory school at large (SNAE, 2016).

LI, like other forms of formal education in Sweden, is publicly funded. However, over the last three decades, Sweden has developed one of the most deregulated, market-oriented education systems in the world (Dahlstedt & Fejes, 2019). This system includes free school choice for parents and students, the right for different actors to establish new schools, and the right to make a profit from publicly funded education. A large number of students in upper secondary school is enrolled at independent schools (26%). While most independent schools are owned by large equity firms, a few are owned by civil society organizations. Only a few independent schools offer the LI programme (Beach & Dovemark, 2019), so LI students are limited in their ability to choose schools for themselves. However, as part of the further development of LI, the Swedish government has allowed municipalities in one county (Östergötland) to try outsourcing the provision of LI to folk high schools (Fejes et al., 2018a).

Folk high schools normally only deliver adult education, directed towards people who are at least 18 years old. Traditionally, these schools (which have a history going back to 1868) were boarding schools where students lived on site. However, today, most students do not live at these schools, and only go there for their studies. Folk high schools deliver a range of different courses—a basic course assisting students to become eligible to enter higher education, plus various vocational and cultural courses. To a large extent, these schools are funded by the State, but they are free to decide which courses to deliver and how to organize them, i.e. there is no national curriculum for folk high schools (Fejes et al., 2018b). The idea behind trials allowing these schools to deliver the LI programme was that this would be beneficial to newly arrived students—not least based on the long history of folk high schools working with students of mixed backgrounds in terms of migration, education, and experience, and based on how well-connected these schools are to other civil society organizations. Furthermore, because folk high schools have a campus-based, social, and safe environment, the argument was that they could potentially enhance the pace at which newly arrived students learned the Swedish language (Ministry of education, 2016).

In this article, we focus on LI, how it is organized for inclusion and what kind of exclusion arises from such an organization. More specifically, we ask the question: in what ways do different settings for LI exclude at the same time as they include?

### Theoretical perspective

In this article, we draw on Lister’s (1990, 2002, 2007) theories of inclusion and exclusion, and specifically her elaboration on the concept of citizenship. Lister (2007) specifically outlines three key dimensions of citizenship: rights and responsibilities, belonging, and participation, each illustrating the individual’s participation in the social community, and thus their social inclusion in society. *Rights and responsibilities* concern the balance between formal and substantive rights (civil, political, and social) and the set of obligations that are tied to membership of a social community. *Belonging* concerns norms, meanings, and identity, including prevalent notions of normality and deviance, i.e. what it means to be a “normal citizen”, compared to being an “outsider” (cf. Yuval-Davis, 2011). Of particular importance is the drawing of boundaries (in the social body, the territory and the population), whereby certain categories of the population are attributed specific values and characteristics, and are thus construed as different from people of other categories. *Participation* concerns the means by which various sections of the population are able to raise their voices and address their claims to change, by actively taking part in formal decision-making processes, in the formal or non-formal activities of civil society, or in their everyday lives.

Inclusion and exclusion are not a matter of being either included or excluded, but should rather be understood as positioning on a continuum, which changes over time. Individuals may be formally included in the societal community in the sense of being granted the formal rights of citizens (civil, political, and social), but at the same time these individuals may be in the position of not really being able to access or substantially exercise these rights (Dahlstedt & Hertzberg, 2007), i.e. they might not be allowed to become full members of the community. Different rights are mutually dependent on one another. For example, access to social rights is important for actually making use of both civil and political rights. Accordingly, deficiencies in social rights, tied for instance, to weak personal finances, low education, residential segregation, or discrimination, are directly related to a lack of political participation (Lister, 1990).

## Methods and data

This article draws on a larger research study consisting of interviews with 74 LI students, 27 teachers and six principals at five different schools. In selecting schools for the study, we wished to gain a sample based on different kinds of organizations that were currently (at the time of the study) organizing LI for asylum seekers aged 16–19. Thus, we selected both regular upper secondary schools and folk high schools. Furthermore, as upper secondary schools in Sweden are provided by both municipalities and independent schools operating on behalf of municipalities, we also included one independent school in the sample. All five schools were located in two mid-sized cities in Sweden. The reason for selecting these cities was because they had the temporary right to deliver the LI programme at folk high schools. Our sample thus consisted of two municipal-run upper secondary schools, one independent upper secondary school, and two folk high schools.

In order to gain an understanding of the activities taking place within the LI programme at the schools, researchers went on-site to conduct observations of teaching and what was happening between classes, as well as conducting informal conversations with students, teachers, and principals/managers. These observations were combined with formal interviews with principals/managers, teachers, and students at each school. All the principals and managers responsible for the LI programme at each school were interviewed, as well as most of the teachers. Teachers were selected based on availability. For student interviews, we wished to gain a wide representation of student backgrounds, in terms of gender, country of origin, and previous educational attainment. However, many of the students interviewed had fled from Afghanistan and Syria, and quite a few had very little experience of the previous schooling. About half of them had already received a resident’s permit, and half were still waiting for a decision.

All the interviews with students were conducted individually in order to provide space for them to further elaborate upon their meaning-making regarding their current situation, background and ideas about the future. Interviews with teachers were conducted as group interviews, with between two and five teachers in each group. Conducting group interviews with teachers had the aim of providing opportunities for a discussion among them about the programme and their teaching. The interviewees were distributed as illustrated in Table I. Interviews with principals and managers were conducted individually as well as the group interviews, depending on whether the specific school had one or two principals/managers responsible for the LI programme. The total number of interviewees in the study is illustrated in Table I.

**Table I.** Number of interviewees

	Students	Teachers	Principals
Municipality-run school 1	17	11	1
Municipality-run school 2	15	4	1
Folk high school 1	9	3	1
Folk high school 2	21	4	1
Independent school	12	5	2
Total	74	27	6

Almost all the interviews were recorded and transcribed verbatim. A few students did not wish to be recorded, and in these cases, notes were taken instead. An interpreter was only used in a couple of student interviews. Rather, students generally wished to be interviewed in Swedish, because for many of them the interview itself became an opportunity to practise the language. Interviews with students range between 10 and 60 minutes, while interviews with teachers and principals were usually longer, lasting up to 90 minutes. All the interview transcripts and field notes from observations and informal conversations serve as the basis for the analysis presented below. The research has undergone appropriate ethical vetting and was approved by the regional ethical committee (Ref. no. 2017/280-31).

Conducting interviews with students in precarious life situations, with implications for their health and well-being, raised several issues for us as researchers. Firstly, should we involve interpreters or not? We deemed it necessary as well as ethically most appropriate to let the students themselves choose whether an interpreter should be present or not. This meant that some interviews were quite short due to limitations in communicating in Swedish. Secondly, what kind of questions should we ask? As our focus was on students’ meaning-making about their current situation and their ideas about the future, we made sure to put questions about their background at the end of the interviews. This decision was made in order to try to avoid going too deeply into their past experiences of being uprooted from their previous countries of residence.

We also identified that both residence status and the students’ age played a role in the interviews. Students who were still waiting for a decision on their asylum application, as compared to those who had gained a resident’s permit, were much more focused on talking about their anxieties and fears, as well as describing their current life situation as highly stressful—as life being on hold. Such anxieties and fears also seemed to be related to the students’ ages, or at least related to those students who themselves said they were 18 or 19. Many of these students expressed fears about having to move on to adult education programmes in order to complete their education, while others were afraid of being expelled from Sweden if they did not finish their education on time. In sum, we could see that many of the students did not feel well, due to various different factors, and thus, as we will illustrate in our analysis, the school had become an important place for them.

In order to analytically gain insight into how LI is organized as a space for both inclusion and exclusion, we have thematically organized our data as follows: Firstly, drawing on Lister’s concepts of rights and responsibilities, participation, and belonging, we have identified how a desire for inclusion emerges in the interviews in relation to these aspects. Secondly, based on this desire for inclusion, we have identified how the different institutional settings differ, and how mechanisms of exclusion are put in place.

### Results

In this section, we present our analysis, which has been guided by the three dimensions of inclusion and exclusion identified by Lister (1990, 2002, 2007): rights and responsibilities, participation, and belonging.

### Rights and responsibilities

Asylum seekers up to the age of 20 are awarded the right to go to school in Sweden. For those aged 16–20, LI is the first programme they enter. However, even though someone might have formal rights, the question is whether these rights are also substantive, i.e. whether the students can fully make use of these formal rights, whether they are *actually* used, and if so, in what ways.

We can see that the teachers in our study are engaged and concerned about students’ attendance at school. Students are not forced to use their rights, and this can result in some of them not attending, or in the end being excluded from participation in school. As this teacher describes it:

If they sit at home and can’t cope. Then things end. And yes, we can unregister students. We have discussed this with some students, that they’ve been absent for so long that we might have to unregister them. But what happens then to the students? There’s nothing else for them. Even though you’re almost constantly absent, these students might attend one or two afternoons a week anyway. And this is a lifeline; [what happens] if we cut that? (Teacher at a municipality-run school)

Here, the teacher explains that there are newly arrived migrants who do not use their formal right to participate in studies. This might be because they do not have the energy due to being in a very precarious life situation. Even though the school and teachers have the right to unregister students who do not attend, this teacher states that the school is so important for these students, a lifeline, that it is better to continue to provide the right to participate, even though such participation is irregular. Another teacher elaborates on a similar topic, when she describes how important the school is for these students, and especially those who are unaccompanied minors.

I also think that it’s important for them to have a stable and safe place to go to. For those having a really hard time and not knowing: “Will I be able to stay or not? Where will I live? When will I have to leave? What will happen to the place where I’m staying?” Then the school is a safe place. It’s here. It’s open five days a week. Even if some students turn up exhausted, from not being able to sleep at night, they still come here. Some of them might sleep during class, but they’re still here, and that’s important. That they feel school is meaningful, that they’re individuals and that we see them. I think this is very important. Particularly for students who are unaccompanied minors, that they have someone to turn to. (Teacher at a municipality-run school school)

Here, the teacher elaborates on the importance of students actually using their right to participate, even when such participation is based only on attendance—“some might sleep during class, but they’re still here”. In sum, these two teachers not only illustrate that these students have a right to participate in studies, but that the teachers are going to great lengths to support them to make substantive use of their right to participate in school.

In the next section, we elaborate further on the issue of participation, in terms of participation as not only a formal right but also a substantive right. Here, LI students are supposed not only to participate as such, but to participate in certain conditions in order for them to develop a feeling of belonging.

### Participation and belonging

Participation and belonging are both crucial dimensions of inclusion, and they are closely linked. Participation might provide space for the development of a feeling of belonging, while a feeling of belonging, in turn, might encourage participation. In our empirical material, we identified how three of the schools (both folk high schools and the independent school), went to great lengths to organize LI education in a way that could potentially encourage the development of a feeling of belonging among LI students.

One organizational form that was used at these schools was to locate the SI classrooms centrally within the school building. By doing so, the ambition was to have students meet across cultural backgrounds. As one of the teachers at the independent school explains:

That’s an idea on our part, to keep them in the middle of the school. When I worked with LI in another school, we had the LI students in temporary buildings. Physically, we were outside of the school, in temporary barracks. And it seems that the students at that school are now placed in the basement. (Teacher at the independent school)

Here, the teacher elaborates on the importance of LI students being fully integrated into the school, where the very location of the classroom is construed as important. At this school, the classroom is located in the middle of the school, right next to the principal’s office. Furthermore, the students have their lockers just outside the classroom, in a space where students from other programmes are also present. The LI classrooms are similarly located in the two folk high schools.

Another strategy that was used to get students to meet across classes involved replacing all classes across all courses at one folk high school, once a week, with different activities aimed at student interaction across courses and classes. As one of the teachers at the school explains:

We have themed days, activity groups once a week when students are mixed [together]. They have a choice, and our ambition is for these activities to create fun meetings. Of course, they [LI students] meet and greet their fellow students on the basic course that I also teach. Unfortunately, the LI students don’t meet many other students at the school. Not to the extent one might wish. (Teacher at a folk high school)

As we can see, these ways of organizing themed days are aimed at getting students to meet across programmes, something that does not otherwise happen very much. The hope, as purveyed by teachers, was that these themed days would “create fun meetings” and provide opportunities for LI students to meet other students. However, as illustrated by both teachers and LI students, such meetings do not seem to happen very often.

Several LI students state that they barely make contact across classes, and they do not really dare to make contact with students from other classes. As two students tell us:

I don’t have any Swedish friends, but I really want some. As we’re in the LI class, we don’t meet many Swedes. There are no Swedes here. In one school I attended previously, there weren’t any Swedes either. (Student at a municipality-run school)

There are Swedes. But we don’t have any contact with them, because we don’t know them. I’ve been here for almost one and a half years. But I haven’t had a single Swedish friend. There is this kind of feeling, but no. I don’t dare talk to them. That’s how it is. (Student at the independent school)

Despite the explicit intention of arranging meetings between students across programmes, such meetings seem quite rare. However, in the interviews, there is a range of examples of meetings and friendships that have developed within the LI class. As this Somali student elaborates:

They also came from Somalia. They do have some problems. They’re ashamed of not being able to speak Swedish. If they use the wrong words, they’re ashamed. But I tell them: ‘You will learn Swedish. It doesn’t matter. You can speak and while doing so, you will learn Swedish.’ (Student at a folk high school)

Thus, by organizing activities across programmes, and by locating LI classes in the middle of the school, a certain kind of belonging is expected to emerge among the LI students. It is expected that belonging will develop based on LI students meeting (Swedish) students from other programmes. However, what is neglected in this line of argument is the kind of belonging that might, and actually does, emerge within the LI classes themselves, as in the example of the LI students with Somali origins above.

Despite the explicit ambition to organize the LI programme in a way that potentially promotes a feeling of belonging among the students, there are also conditions that instead promote the opposite. This will be further elaborated in the next section.

### Mechanisms of exclusion

Compared to the folk high schools and the independent school, the municipality-run schools organize their LI classes more off to the side, away from the other programmes at these schools. At one of these schools, the LI classes have their classrooms in three corridors that are a little separate from the rest of the school building. Very few other students, besides the LI students, pass through these corridors. During breaks, the LI students go to the cafeteria, where students from other programmes also congregate. Apart from this, the LI students mostly keep to their “own” corridors, waiting for the lessons to start while looking at their phones or chatting with each other.

At the other municipality-run upper secondary school, LI classes have their classrooms in an older building separate from the rest of the school, which was previously used for industrial vocational programmes. The building is old and tired, the walls are made of concrete and the windows are high up, making it impossible to see out of them. The rooms are lined with electric sockets as a reminder of their previous incarnation as computer rooms (and, before that, workshops). In the interviews, students repeatedly complained that the toilets are dirty and disgusting. During breaks, they were consigned to the stairwells in the main building, where some sofas and chairs were placed. There was also a recreation room in the basement.

During the winter, extra radiators had to be placed in the classrooms to keep them warm and most of the students needed to keep their jackets on to avoid getting too cold. For a few weeks in February, when temperatures were close to minus 10 degrees Celsius, each student had to use a fan heater to keep their hands warm. At one point, issues regarding the poor facilities led the teaching to be moved into the main building.

The poor standard of the classrooms was frustrating for students and teachers alike. “We’ve taught there for two years now,” one teacher told us. According to the teachers, the placement of LI students in a separate, run-down building helps to shape a feeling of being “other” among the students. “It’s really sad that we’re so separated,” the teachers tell us. “If we’d been located in the main building, our students might have felt they were actually on their way to becoming eligible to enter a national programme.” Another teacher tells us:

They just want to be in a regular Swedish class with Swedish students, they tell us. Now they think: ‘Here, we’re all migrants. We don’t learn anything from each other because no one knows Swedish. If we were in a regular Swedish class, we would … ’ (Teacher at a municipality-run school)

Let us take one concrete example of what the mechanisms of exclusion, however unintentional they might be, might look like at this particular school. One day, the school management decided to treat the students to coffee and buns. Information was sent to students on all the national programmes, but not to the LI students. This upset the LI students, who wondered why they had been excluded. “Is it because we’re migrants?” one student asked us during the interview. The students went over to the main building anyway, to get some coffee and buns. With the help of their teachers, some students took the initiative to draw up a written petition to be delivered to the principal. This example illustrates how LI students are physically excluded at this school, by being put in an old building away from the main, newly renovated one. But they were also forgotten in relation to an activity aimed at the entire school. However, this example also illustrates how, in response to such treatment, the students were galvanized into demanding change in order to become included as full members of the school.

However, as we will illustrate in the next section, no matter which of the schools the students attended, they were positive, and school was construed as meaningful.

### A space that might support the development of belonging?

In our analysis, we can see that all educational settings are experienced as meaningful places of stability by the LI students, in an otherwise quite chaotic and stressful life situation, particularly for those students who do not yet have a residence permit. Holidays are especially stressful for them, because school is then closed. Students are generally positive towards their teachers, and describe them using words such as “great” and “fantastic”. Students find teachers to be “good at explaining”, “they care about me”, “they tell us we do well”, “that we will manage”, and “teachers are fun, they make a lot of jokes”. For many students, teachers emerge as important people. As one student tells us:

Johanna is really nice. She listens to the guys, she makes me happy. When I had a problem, she helped me. And she helps the guys a lot. Madeleine is also very nice, and she listens a lot. She’s nice to everyone. As is Georgios. (Student at a folk high school)

Such a description is confirmed by the teachers themselves, who feel humble about teaching these students. As one teacher says:

We’re grateful for this. They will contribute to society. If they’ve arrived in this place, they will be successful later on. (Teacher at the independent school)

Another teacher says:

We’re grateful to have been given this assignment. It’s the most interesting thing we’ve done lately. (Teacher at the independent school)

Our observations at all the schools confirm this picture. Students often seek contact with teachers after the lessons end, in order to discuss different issues. Teachers’ engagement often goes beyond what could be expected of them as teachers. We have seen examples of teachers helping students to pay for bus tickets, and putting them in contact with sports coaches. However, such engagement has a downside: it can be psychologically quite hard for teachers to support students in such vulnerable positions, which may eventually lead to teachers burning out. Seeing some of their students having their residence permit application turned down is especially hard on teachers. In our interviews, many teachers tell us how their relationships with students raise a pedagogical dilemma: on the one hand, they need to be present and support students, and on the other hand, they need to avoid becoming too emotionally involved (see Fejes et al., 2018a).

In sum, school emerges as an important space for students, with teachers across all the schools providing support and engagement. Thus, students’ descriptions of school as an important space also confirm the way in which teachers talk about the great importance of school for LI students, particularly unaccompanied minors.

## Discussion

In this article, we have illustrated how the LI programme for newly arrived migrants aged 16–19 years simultaneously both includes and excludes. More precisely, by drawing on Lister’s (1990, 2002, 2007) understanding of inclusion and exclusion, we have been able to identify how, on the one hand, LI students are offered the right to education, but, on the other hand, they do not always have substantive means of actually making use of this right, not least due to a precarious and stressful life situation at large. However, teachers are working hard to get students to come to school because this is construed as important in order for students to develop a feeling of belonging. At school, certain organizational methods are used to promote the development of such belonging. Among others, this includes placing the LI classrooms in the middle of schools, and organizing activities across the entire school, in which students from different programmes are expected to meet. However, these ways of organizing school might make invisible the kind of belonging that also develops *within* the LI classes. At the same time, we have identified organizational arrangements that more directly exclude students, rather than including them. At some schools, these involve locating students in classrooms outside the main buildings, or forgetting to invite LI students to general school activities. However, no matter what kind of organization or conditions were identified at the different schools examined, students are generally quite positive. They find their education both meaningful and supportive, both in terms of the educational process itself, and also as a space for social support, in a precarious life situation.

Our analysis partly concurs with previous studies on newly arrived students in upper secondary school, particularly in how they feel “othered” by being placed in specific classes, or to one side of the main activities, in Sweden (cf. Nilsson Folke, 2017; Sharif, 2017; Skowronski, 2013), and in other countries (cf. Terhart & Devitz, 2018; Torbjørnsen Hilt, 2016). We can relate here to how, in his study of newly arrived students in Sweden, Sharif (2017) describes the formation of ethno-cultural spaces in the school, whereby newly arrived students are excluded, both physically and mentally.

Our results also provide some new insights into the organizing of LI, at least in Sweden. By focusing on five different schools, representing different institutional organizations in the Swedish educational landscape, we have been able to identify differences in how inclusion and exclusion operate. As illustrated, the space is organized in ways that separate newly arrived students from other students at both of the municipality-run upper secondary schools, but not at the folk high schools or the independent school. In these latter schools, newly arrived students have their classrooms centrally located within the school. Even though we cannot draw conclusions on a more general level concerning differences between schools, our results raise interesting questions. Why is it that there is such a difference in our material between schools? One potential answer is that both folk high schools and the independent school are rather small compared to the municipality-run schools. Thus, the LI students are not as anonymous as might otherwise be the case. Another potential answer would be that the folk high schools and the independent school are value-based, with a specific emphasis on the relational dynamics of organizing teaching and learning. Thus, a focus on issues regarding the organization for inclusion might be more prominent.

Summarizing our argument, the question of inclusion and exclusion in school, as well as elsewhere, is not a matter of either/or. Rather, these processes coexist simultaneously. This is particularly visible in our example of how the placement of LI classes outside of the main building was combined with an ambition among teachers to support students in their learning. Nor do inclusion and exclusion involve only one isolated dimension, but several interrelated ones, such as the three that are the focus of this article: rights and responsibilities, participation, and belonging. For example, even though education may exist as a formal right, for newly arrived asylum seekers as for everyone else, this right is not necessarily accessible or utilized. This is influenced by a range of factors, among which are existing social conditions. Participation in the school is thus enabled by the social conditions of the students. In turn, in a range of different ways, participation creates possibilities for the development of feelings of belonging, not least in terms of building a sense of being seen, listened to and thus recognized as a human being. Such belonging may take different forms, of which some may be construed as valuable (i.e. those based on meetings between LI students and native Swedish students), others as problematic (i.e. those based on meetings within the category of LI students). These processes of inclusion and exclusion are complex, and often contradictory. Thus, in order to create an understanding of school as a place for inclusion (and exclusion), we need to take a broad approach that makes it possible to identify these different dimensions, and their relations.
